# PREVIEW Behavior Modification Intervention Toolbox (PREMIT): A Study Protocol for a Psychological Element of a Multicenter Project

**DOI:** 10.3389/fpsyg.2016.01136

**Published:** 2016-08-10

**Authors:** Daniela Kahlert, Annelie Unyi-Reicherz, Gareth Stratton, Thomas Meinert Larsen, Mikael Fogelholm, Anne Raben, Wolfgang Schlicht

**Affiliations:** ^1^Division Exercise and Sports, University of Education Schwäbisch GmündSchwäbisch Gmünd, Germany; ^2^Chair Exercise and Health Science, Stuttgart Research Initiative Human Factors in Ageing, Technology, and Environment, University of StuttgartStuttgart, Germany; ^3^Applied Sport, Technology, Exercise and Medicine Research Centre, Swansea UniversitySwansea, UK; ^4^Department of Nutrition, Exercise, and Sports, University of CopenhagenCopenhagen, Denmark; ^5^Department of Food and Environmental Science, University of HelsinkiHelsinki, Finland

**Keywords:** behavior modification, overweight, obesity, physical activity, diet, type-2 diabetes, theory- and evidence-based

## Abstract

**Background:** Losing excess body weight and preventing weight regain by changing lifestyle is a challenging but promising task to prevent the incidence of type-2 diabetes. To be successful, it is necessary to use evidence-based and theory-driven interventions, which also contribute to the science of behavior modification by providing a deeper understanding of successful intervention components.

**Objective:** To develop a physical activity and dietary behavior modification intervention toolbox (PREMIT) that fulfills current requirements of being theory-driven and evidence-based, comprehensively described and feasible to evaluate. PREMIT is part of an intervention trial, which aims to prevent the onset of type-2 diabetes in pre-diabetics in eight clinical centers across the world by guiding them in changing their physical activity and dietary behavior through a group counseling approach.

**Methods:** The program development took five progressive steps, in line with the Public Health Action Cycle: (1) Summing-up the intervention goal(s), target group and the setting, (2) uncovering the generative psychological mechanisms, (3) identifying behavior change techniques and tools, (4) preparing for evaluation and (5) implementing the intervention and assuring quality.

**Results:** PREMIT is based on a trans-theoretical approach referring to valid behavior modification theories, models and approaches. A major “product” of PREMIT is a matrix, constructed for use by onsite-instructors. The matrix includes objectives, tasks and activities ordered by periods. PREMIT is constructed to help instructors guide participants' behavior change. To ensure high fidelity and adherence of program-implementation across the eight intervention centers standardized operational procedures were defined and “train-the-trainer” workshops were held. In summary PREMIT is a theory-driven, evidence-based program carefully developed to change physical activity and dietary behaviors in pre-diabetic people.

## Introduction

The incidence of Type 1 or Type 2 Diabetes Mellitus (T2D) is increasing worldwide (NCD RisC, 2016a). For example the number of adults age 55–74 years with T2D is set to double by 2025 (compared to from 2000; WHO, 2014). Brinks et al. (2012) point out that in order to prevent one million cases of T2D approximately 90% of all people with pre-diabetes have to participate in interventions aiming to reduce the transition from pre-diabetes to diabetes.

Overweight and obese people have a high risk for T2D. Despite all efforts to stop overweight and obesity, worldwide prevalence is increasing (Stevens et al., 2012; NCD RisC, 2016b). Being overweight [defined as a Body Mass Index (BMI) of 25 kg/m^2^ to 29.9 kg/m^2^] increases the risk of T2D 5-fold compared to normal weight (a BMI of 21 kg/m^2^) in women (Colditz et al., 2005). Even a BMI at the end of the normal weight range (i.e., 23 kg/m^2^ to 24.9 kg/m^2^) is associated with a considerably higher risk than a BMI less than 23 kg/m^2^ (Hu et al., 2001). Moreover, T2D is an insidious disease, starting with pre-diabetes, which is defined as impaired fasting glucose and impaired glucose tolerance. Being pre-diabetic increases the risk of getting T2D by nearly a fifth (Saaristo et al., 2005).

Preventing diabetes is key if the upward trend in its prevalence is to be halted. There is evidence that a 5–10% weight loss can improve the health related risk status of overweight and obese people (National Institute for Health Clinical Excellence, 2006). Intervention studies, such as the Finnish, the US and the Chinese Diabetes Prevention Studies, have been developed to effect behavior change, targeted to national cohorts in the respective countries (The Diabetes Prevention Program (DPP) Research Group, 2002; Lindström et al., 2006; Li et al., 2008).

It is important to recognize that changing lifestyle behaviors to effect weight loss is difficult. Moreover, overweight and obesity are chronic relapsing conditions (Stubbs et al., 2011) and most people regain weight after initial weight reduction (Meinert Larsen et al., 2010). Inactivity and poor dietary behavior are proximate risks for overweight and obesity and both are affected by an interaction of personal (e.g., attitudes) and environmental (e.g., unhealthy food choices) conditions. The obesogenic environment also makes unhealthy behaviors easy (Egger and Swinburn, 1997) resulting in risk type behaviors which are stable over time and often guided by routines (Aarts and Dijksterhuis, 2000). In this environment situational cues trigger specific risky behaviors (e.g., “snacking while watching TV”). Even if people intend to change their habits they often only succeed during the first attempt subsequently relapsing to their former behavior (Bock et al., 2001). Practically, sustaining a reduced weight is challenging and often susceptible to failure.

Nevertheless, systematic reviews (e.g., Avery et al., 2012; Pillay et al., 2015) have revealed important clinical or beneficial practical effects. Although converting these into real world interventions is a complex task (Tricket and Ryerson Espino, 2004). One reason is that behavior is multi-determined and interventions cannot target all mechanisms. There is no “one size fits all”-approach to modifying risky behavior or achieving long-lasting behavior change (Michie et al., 2009b). There is consensus, that theory-driven and evidence based behavior change programs should target the behavior that needs to be addressed (e.g., Hardeman et al., 2005; Michie et al., 2009b, 2013; Avery et al., 2012). Theory-driven assumptions are specifying why a given intervening component or activity will cause behavior change under given conditions (Hardeman et al., 2005; Michie and Prestwich, 2010; Lacouture et al., 2015). “Evidence-based” means that components and activities are effective in changing people's behavior. The literature holds systematic reviews and meta-analyses that have summarized scientific evidence to support behavior change programs. Besides these two criteria, Michie et al. (2013) postulated as further requirements to develop a cumulative science of behavior change, comprehensive descriptions of interventions in research protocols and identifying effective components of behavior change interventions.

A main objective of implementation science is to develop the most effective approach to answer a research question derived from a public health problem. Four steps in translational research were recently distinguished (Lobb and Colditz, 2013): (T1) case series and efficacy trials, (T2) effectiveness studies, developing clinical guidelines, systematic reviews, (T3) effectiveness studies, developing implementation guidelines and (T4) use of evidence based interventions and implementation strategies in the real world. Proctor et al. (2009) distinguish between research for “dissemination” and research for “implementation.” Research for “dissemination” refers to the targeted distribution of information and intervention materials to a specific public health or clinical practice audience. Research for “implementation” tries to find out the best use of strategies to implement evidence-based health interventions within specific settings.

Given these distinctions, the protocol here outlines the PREview behavior Modification Intervention Toolbox (PREMIT) as a theory-driven and evidence-based approach targeted to change the behavior of people at risk for T2D, applicable for interventions in the real world, as it is represented in steps “T3” and “T4”. PREMIT is designed to be adaptable in different countries. It aims at contributing to the required necessities in science of behavior change: (a) to develop theory-driven and evidence-based interventions, (b) that are described in protocols and c) to identify effective components of behavior change interventions. This goes beyond the above cited intervention studies that most often describe the overall protocol of the intervention and lack to describe the behavioral part in detail.

## Methodological approach

PREMIT is part of a project called PREVention of diabetes through lifestyle Intervention and population studies in Europe and around the World (PREVIEW). The PREVIEW project aims to identify an effective way to prevent T2D in pre-diabetics by gathering evidence from population studies as well as a multicenter randomized controlled trial (RCT). PREMIT is a component of the RCT, which investigates the effectiveness of two particular diets and two different intensities of physical activity in order to maintain weight loss in a sample of pre-diabetics (Fogelholm et al., 2013; Raben et al., 2013). The PREVIEW RCT is a multicenter study organized in eight countries worldwide (Australia, Bulgaria, Denmark, Finland, Spain, Netherlands, New Zealand, and United Kingdom). All RCT participants are counseled to change their physical activity and dietary behavior independent of their belonging to one of the specific RCT-arms. The instructors at the study centers use PREMIT to support PREVIEW participants to begin and to stick to a diabetes preventing lifestyle (i.e., being physically active and following a particular diet). All centers obtained ethics approval for the intervention program by their local ethics committees. The trial is registered at clinicaltrials.gov under ID NCT01777893 and founded by the European Union Seventh Framework program (FP7/2007–2013) under grant no. 312057.

**Figure 1:**
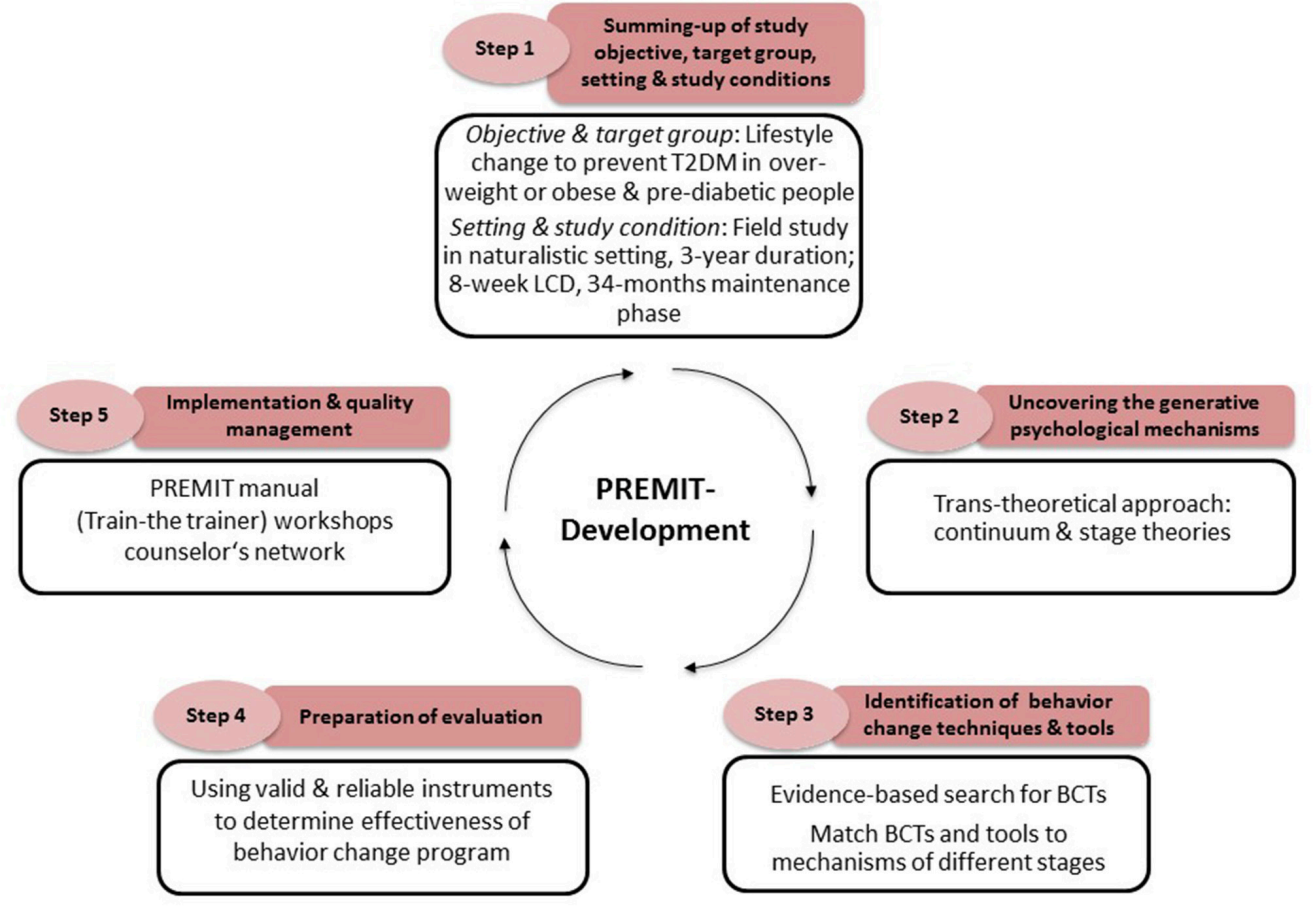
**Stepwise and systematic approach for conducting the PREMIT intervention**.

Designing and conceptualizing PREMIT adhered to the common steps of the Public Health Action Cycle (Rosenbrock, 1995) and the recommendations of the UK Medical Research Council (Craig et al., 2008). PREMIT was mainly developed by three exercise and health scientists in cooperation with scientists responsible for the general study design of the PREVIEW project (i.e., nutritionists, obesity researchers, exercise scientists). The design and conceptualization of PREMIT followed five steps, depicted in Figure 1.

### Step 1: study objective, target group, setting, and study conditions

For the PREVIEW intervention study, a total of 2348 men and women were recruited consecutively. Eligible were people aged 25–70 years, overweight or obese (inclusion criteria is a BMI ≥25.0 kg/m^2^), and pre-diabetic. Pre-diabetes is defined as either having a fasting venous plasma glucose concentration of 5.6–6.9 mmol/l or a venous plasma glucose concentration of 7.8–11.0 mmol/l at 2 h after oral administration of 75 g glucose with fasting plasma glucose less than 7.0 mmol/l.

PREVIEW is constructed as a randomized, clinical intervention, taking place in the natural setting (Fogelholm et al., 2013; Raben et al., 2013). The overall objective of PREVIEW is to prevent T2D in those at risk. The behavior modification intervention in PREVIEW aims to reduce the T2D risk through lifestyle changes in dietary behavior and physical activity (i.e., to change the macronutrient composition of food and to become physically active). During the 36 months intervention participants are encouraged to reach and to maintain a predetermined volume of physical activity. They should also follow one of two diets. Participants were randomly assigned to the experimental conditions. In the first phase of PREVIEW participants start with an 8-week low calorie diet (LCD). PREVIEW use the Cambridge Weight Plan (see: http://www.cambridgeweightplan.com), which is a diet formula. The plan provides enough protein to protect lean tissue, delivers the right level of carbohydrate and the right levels nutritional components (i.e., vitamins, minerals, etc.) to maintain health. Initially meal replacement supplements are given in form of porridges, shakes and soups. They combine all necessary food groups to satisfy a body's nutritional needs. Cambridge weight plan is intended to stop a person's craving foods. Gradually solid foods are introduced into the meal plan. The goal of the LCD phase is to achieve an eight per cent weight loss. Participants who achieve this are then randomly assigned to one of 4 groups in the 34-month behavior change phase where they follow PREVIEW specific diet and physical activity regimen (see Fogelholm et al., 2013; Raben et al., 2013). To support their lifestyle changes all participants take part in 17 group visits where they are counseled by a health professional (i.e., dieticians, physical activity instructors). The main contents of group visits were described in a manual and structured in PREMIT (see Supplementary Table 1). Onsite instructors were trained before the intervention started. Instructors met monthly in a telephone conference to discuss salient issues, share best practice and support each other. At any given date one representative of the PREMIT development team from the University of Stuttgart joins the instructors telephone conferences (see Step 5).

### Step 2: uncovering the generative psychological mechanisms

A systematic literature search was done using standard electronic databases (Cochrane Library, PsycNet, PubMed, Web of Knowledge) to detect those mechanisms which could be modified and should be addressed to motivate the PREVIEW participants to change their lifestyle. In line with Bauman et al. (2002) and Michie and Prestwich (2010), we searched for those mechanisms matching the requirements specified in the intervening conditions of PREVIEW. The selection was based on evidence from systematic or meta-analytic reviews (Michie et al., 2009a; Greaves et al., 2011; Williams and French, 2011; Olander et al., 2013) by examining the reported effect sizes (e.g., d, r_g_).

One source of uncovering mechanisms are well-established theories in health psychology. Theories, models or approaches used in behavior change are usually construed as an intended and planned decision-making process. Most of the behavior modification theories share the following assumptions: Behavior change will more likely occur if a person perceives themself as vulnerable (vulnerability) suffering from a severe disease (severity) and anticipating a behavior that will reduce the risk (outcome expectancies). A further critical variable is “self-efficacy” (Bandura, 1977). For instance, people differ in how much they believe in their abilities to reduce the risk of T2D by following a recommended dietary and physical activity behavior. Self-efficacy is a crucial variable to make health behavior change probable and sustainable in general (Bauman et al., 2012) as well as in people suffering from T2D (Strychar et al., 2012). According to behavior-change theories, the psychological variables are the “adjusting screws to fine-tune” or the mechanisms making behavior change more likely and successful.

According to stage theories (we refer here to the Transtheoretical Model by Prochaska and DiClemente, 1992), behavioral change follows a stepwise process, whereby different variables are relevant in particular stages. Stage theories distinguish at least four stages starting with a stage of unconsciousness, where vulnerability is hidden, followed by stages of consciousness, where the risk of the behavior becomes obvious and over a number of further steps ending with a stage where the new healthy behavior is habituated (Prochaska and DiClemente, 1992).

Stage-based approaches assume that behavior change is more likely if an intervention is tailored to the needs and expectations in the respective stages in order to make stage transition more likely (Prochaska and DiClemente, 1992). For instance, people who are not aware of their risky behavior should become aware of it. Fear-appeal is one way to call a person's attention to their risky behavior by showing frightening pictures about consequences of T2D. In a meta-analysis fear appeal was effective in making people aware of the severity of a disease and their own vulnerability although actional self-efficacy was also important in those willing to change (Witte and Allen, 2000). Having already started with the new behavior, *coping self-efficacy*, which means believing in the ability to master difficult or stressful situations, becomes relevant (Schwarzer and Renner, 2000).

Combining different theoretical approaches is quite common and useful in developing interventions (Ogilvie et al., 2011). PREMIT follows a trans-theoretical approach, by using well-established theories of health behavior change. In particular the compatibility of the theories, models or fragments were selected, to guide the intervention by making it theory-driven. A stage-oriented approach is used to tailor the intervention to different phases of behavior change (Prochaska and DiClemente, 1992). Further, PREMIT was oriented toward the core constructs and predictors from continuum theories such as the Health Action Process Approach (Schwarzer, 2001), the Social Cognitive Theory (Bandura, 1996), the Self-Determination Theory (Deci and Ryan, 2008), Habit Theory (Wood et al., 2005), Goal Adjustment Theory (Wrosch et al., 2003) and the Theory of Learned Optimism (Seligman, 1998). PREMIT distinguishes 4 stages matched to the phases of the RCT (see Step 1): (1) Preliminary stage (LCD phase), (2) preparation stage, (3) action stage and (4) maintenance stage. The duration of each phase was based on the stages of change (Prochaska and DiClemente, 1992), on experiences gained in prior health behavior change interventions as well as on the overall study protocol (see Step 1).

The premise of PREMIT is that in order to change the behavior of the participants, their psychological states have to be addressed and changed by applying the most suitable intervention components (see Step 3). The psychological guidance of study participants in PREVIEW started during the *preliminary 8 weeks LCD stage*. The main objective of stage 1 is to convince participants that they are at risk (vulnerability) of suffering from a severe disease (severity), but that they will have a good chance to prevent the onset of T2D if they change their behavior (outcome expectancy) after losing weight and adhering to this lifestyle in the long run. In the preliminary stage participants were encouraged to lose at least eight per cent of body weight by adhering to the prescribed LCD.

The *preparation stage* lasts four consecutive weeks. The main goal of the group visits is to motivate participants to start the recommended diet and physical activity guidance. At this point counselors emphasize strong commitment to participants' behavioral goals (intention), favorable beliefs about the consequences of behavior change itself (outcome expectancies) and the ability to follow the recommended behavior (self-efficacy).

The *action stage* lasts for 14 weeks. The face-to-face contact between participants and counselors fades out during this period. This is one reason why the main goal during the action stage is to help participants stick to the recommended behavior, autonomously. Self-regulation and self-control skills are also important during this stage, for example monitoring behavior, resisting temptations and concurrent motives and, adjusting behavioral goals in a beneficial manner (goal adjustment).

After 6 months participants reach the *maintenance stage* which lasts for 130 weeks (2.5 years). Six months is a critical period in behavior change interventions as participants who adopted a new behavior lapse to their former habits (e.g., Bock et al., 2001; Kwasnicka et al., 2016). Thus, the main aim of this stage is to prevent lapses and relapses. PREMIT directs counselors to help participants learn to cope with difficult situations (coping self-efficacy) and resume the prescribed behavior even if lapses occur.

All modifiable mechanisms identified for the PREMIT intervention and their sources of reference (theory) as well as the allocation to the different stages are provided in Table 1.

**Table 1:** **Predictors and stages of behavior modification in PREMIT as well as their source of reference in brackets**.

**Predictor**	**Phase**	**LCD^a^-phase (week 1-8)**	**Preparation stage (week 9–12)**	**Action stage (week 13–26)**	**Maintenance stage (week 26–156)**
		**(founded by Transtheoretical Model; Prochaska and DiClemente, 1992)**			
Intention (Health Action Process Approach (HAPA); Schwarzer, 1992)					
Outcome expectancies (HAPA; Schwarzer, 1992; Social-cognitive-theory (SCT); Bandura, 1996)					
Actional self-efficacy (HAPA; Schwarzer, 1992; SCT; Bandura, 1996)					
Social support (SCT; Bandura, 1996)					
Self-determination of motivation (Self-determination theory; Deci and Ryan, 2008)					
Temptations (Habit theory; Wood et al., 2005)					
Habit strength (Habit theory; Wood et al., 2005)					
Coping self-efficacy (HAPA; Schwarzer, 1992)					
Goal adjustment (Goal adjustment theory; Wrosch et al., 2003)					
Attribution (Theory of learned optimism; Seligman, 1998)					

a *LCD, Low calorie diet*.

### Step 3: identification of behavior change techniques and tools

The identification of mechanisms is not sufficient in order to develop an effective behavior modification program. The question is, how could these mechanisms be influenced to change participants' lifestyle? “Behavior change techniques” (BCT; Michie and Johnston, 2008) are developed and tested to solve the problem. The CALO-RE taxonomy by Michie et al. (2011) describes 40 different BCTs^1^. Based on an expert consensus process on the effectiveness and applicability of the BCTs, those relevant to PREMIT were chosen according to empirical evidence supporting lifestyle changes in overweight and obese people (e.g., Williams and French, 2011).

The main part of work and discussion in this step was done at a round table workshop lasting 4 days. Five experts experienced in the field of health behavior and behavior change took part in this workshop. As a result BCTs were classified based on the strength of evidence that they could change behavior. Subsequently, tools for applying BCTs were identified from the respective research report or selected based on expert opinion from corresponding interventions. For instance, “barrier identification and problem solving” could be delivered by counselors through the use of “mental contrasting” techniques (Kappes and Oettingen, 2014).

The mechanisms, linked to the respective stages, BCTs and appropriate tools, were aligned with each stage of PREVIEW and collated in the PREMIT toolbox (see Supplementary Table 1). The matrix includes information about the behavior change stage (column 1), the stage-specific goals (column 2) that are related to the respective behavioral mechanisms (column 3), group visits (column 4) in which this “topic” should be targeted and the behavior change techniques and tools (columns 5 and 6) that should be applied. Further, the matrix specifies the respective assessments (column 7) that are necessary for evaluating the intervention (see Step 5).

In some cases the same mechanism is addressed using more than one BCT both within and between intervention stages. For instance, during the preliminary LCD stage, participants are asked to lose weight but they are not required to change lifestyle behaviors. Hence, in such a stage, actional self-efficacy (mechanism) could be enhanced by prompting people's focus on their past success. One way to do this is to write down past successful weight loss episodes (e.g.,: “I have lost x% of body weight before, so I can do it again”). As soon as participants start to change their behavior (in month 2 of the PREVIEW project), providing feedback using a physical activity log or a dietary compliance questionnaire would promote participants' action self-efficacy.

BCTs support all mechanisms included in PREMIT with the exception of “attribution theory” (Theory of Learned Optimism; Seligman, 1998). Attributions refer to how people explain (negative or positive) behavioral occurrences. This is particularly relevant when trying to resume the recommended lifestyle after a lapse. Attributions could be more or less beneficial (e.g., “I always fail to reach my goals” vs. “This is because I had a busy schedule this week”). It is important to counsel participants toward beneficial attributions (Seligman, 1998).

### Step 4: preparation of evaluation

PREMIT guides the intervention program and product evaluation. Following the recommendation of the Medical Research Council (Craig et al., 2008) the *effectiveness* of the PREVIEW trial will be evaluated by means of different outcome variables. The primary outcome in PREVIEW is the incidence of T2D at the end of the intervention. The goals to reach this objective are to lose weight and to maintain weight loss by motivating the participants to follow a special kind of diet and to reach a prescribed volume of physical activity. Therefore, one criterion for effectiveness of the behavior change program is the participants' lifestyle at the end of the intervention period. As behavior change is determined by social-cognitive mechanisms like self-efficacy, outcome expectancy and other mechanisms integrated in PREMIT, changes of these mechanisms are a further criterion to evaluate the effectiveness of PREMIT. Measuring these changes will be done in a repeated measures design using valid and reliable measurement instruments to detect changes and dynamic processes (Renner et al., 2012) at six measurement points (e.g., at the end of each behavior change stage; see Step 2) over the 36 months intervention. Mechanisms and behavior will be measured by different means (e.g., accelerometers, questionnaires, and diaries). Since the PREVIEW study is carried out as a multi-center trial in six European and two overseas cities or regions, questionnaires to assess the variables as well as materials had to be available in English and mother tongue of each respective country (i.e., in Bulgarian, Danish, Dutch, Finnish, and Spanish). The translation was done by an iterative process starting with a translation from the English original version of the instruments to the national languages and followed by a back-translation into English. This process was repeated until the translation was of sufficient quality (Brislin, 1970). All questionnaires are provided via an online questionnaire delivery platform, supplied by the PREVIEW partner NetUnion.

### Step 5: implementation and quality management

The final step covers all relevant aspects about PREMIT's implementation and quality assurance. The main goal of Step 5 is to ensure that the intervention was implemented with high fidelity and similarly at each of the eight study centers. For example, one challenge in the implementation process was that counselors had different professional backgrounds and experiences. To overcome this, standard operational procedures (SOPs) were written and training workshops conducted to standardize delivery. In the SOP written for all counselors, declarative knowledge is provided on how to support participants' behavior change goals for diet and physical activity. The SOP entails all relevant background information about behavior modification itself, the modifiable mechanisms, the BCTs as well as the related tools/procedures. A description how to organize each group visit is also provided. This description includes a checklist (i.e., what the counselor has to prepare to make the group visit work), the specific goal of a given group visit (e.g., to promote participants' actional self-efficacy), the respective technique (e.g., prompt focus on past success), the respective procedure (e.g., persuasion) and—if necessary—templates (e.g., a template for participants' self-contracting). An example of a group visit-description is provided in Table 2.

**Table 2:** **Example of a description of a group visit in the PREVIEW study**.

**Group visit 2: LCD period, week 2; suggested duration of group visit: 90 min**	
Checklist	Register body weight for all participants Register if participants report (changes in) AE^a^s or concomitant medication Provide LCD^b^ sachets to participants Ask participants about their experience with the LCD^b^. Encourage them to reflect on the previous 2 weeks and to persist despite possible difficulties
Goal 1	Sensitize your participants to change their habitual behavior
Technique 1	Provide information on consequences of diet and activity behavior in general. Make it clear that type-2 diabetes is a severe chronic disease and that your participant belongs to a group of persons extremely vulnerable to getting diabetes
Technique 2	Information on consequences: Involves information about the relationship between the behavior most of your participants are likely to show and the possible or likely consequences of suffering from type-2 diabetes, usually based on epidemiological data (in the LCD^b^ phase not personalized for the individual)
Technique 3	Fear appeal: Involves presentation of risk and/ or mortality information relevant to the behaviors and images designed to evoke a fearful response (e.g., diabetic foot)
Tool	Persuasive communication (pc)
	Persuasion is defined as a process in which one convinces another person to change their attitudes and behaviors voluntarily
How to?	Explain to participants about the likelihood of becoming type-2 diabetic. Show participants the consequences of type-2 diabetes (e.g., charts, pictures or the like). Use pictures and other materials to induce fear. Use persuasion to convince participants
Goal 2	Promote participants' action-self-efficacy
Technique 1	Prompting focus on past success: Involves instructing the person to think about or list previous successes in performing the recommendations for weight loss and the forthcoming recommendations for behavior change (or parts of it) (before PREVIEW)
Tool	Persuasion
How to?	Provide a prepared sheet in which your participants can write down past successes. Instruct them to think about positive episodes or attempts at dieting and exercising

a AE, adverse events;

b *LCD, Low calorie diet*.

At the beginning of the PREVIEW project, two representatives from each site were trained in a 2-day workshop led by the University of Stuttgart. One workshop was executed face to face in Stuttgart for the European partners and another one was executed as a videoconference for the overseas partners (Auckland, NZ and Sydney, AUS). The workshop attendees learned about PREMIT, BCTs, tools and procedures and how to apply them. Different kind of presentations and role-plays were used. In order to assure quality, a resource pack containing all relevant information about the workshop issues was provided. Complex BCTs were recorded. The video material was supplied to all study centers afterwards.

At the different study centers, the representatives trained all staff members before the PREVIEW intervention trial started. In addition, an instructors-network was established. All PREVIEW instructors and the conductors of the behavior modification program are part of the network. Upcoming questions or issues that are related to the behavior modification program are discussed during monthly network teleconferences in order to share best practice.

## Materials

Standardized written and video material was developed to ensure implementation fidelity of PREMIT at all PREVIEW sites. These were summarized in a SOP that was also used as instruction booklet and work of reference. All the contents of the train-the-trainer workshops were summarized and made available to centers in a written report. Further, video materials were also produced, including examples on how to apply the behavior change techniques during group counseling sessions. Also educational materials and templates for participants were provided for the instructors to use at respective group sessions. These included templates for auditing the environment, concluding a self-contract, formulating SMART-Goals, specifying an action plan and working on barrier management. Further, participants also used a physical activity and dietary log to help self-monitor their week-to-week behavior. Questionnaires to assess the psychological mechanisms triggering behavior change and the behavior itself were also used in the evaluation of PREVIEW (Table 3).

**Table 3:** **Overview of assessments that will be used in order to evaluate the behavior modification program**.

**Mechanisms (variables)**	**Questionnaire**
Social support for diet and exercise	Social support for diet and exercise scales (Sallis et al., 1987)
Habit strength of physical inactivity and poor diet	Habit strength measure (Wood et al., 2005)
Intention	Self-constructed items (adapted from Renner and Schwarzer, 2005)
Actional Self-efficacy (physical activity and nutrition)	The nutrition self-efficacy scale and The physical exercise self-efficacy scale (Schwarzer and Renner, 2005)
Causal attributions (for weight outcomes)	Attributional weight outcome scale (Brubaker, 1988)
Self-regulation goal adjustment	Goal Adjustment Scale (Wrosch et al., 2003)
Coping self-efficacy	Coping self-efficacy for physical activity and healthful nutrition (Schwarzer and Renner, 2000)
Outcome expectancies	Outcome expectancy of behavior change (Subscale for change of nutrition habits and subscale for exercise (Renner and Schwarzer, 2005)
Self-regulation of motivation	Treatment self-regulation questionnaire for diet and exercise (TRSQ; Levesque et al., 2007)

## Results

Our protocol describes a theory-driven and evidence-based behavior modification program in its stepwise development. PREMIT is targeted for overweight, obese and pre-diabetic people. PREMIT is a tool to support PREVIEW instructors help participants to change their lifestyle. The objective of this lifestyle change is the prevention of weight-regain and subsequent T2D.

PREMIT is based on five closely related, progressive steps. These steps are driven by theoretical knowledge, based on empirical evidence and adaptable to each center's local context of the multicenter randomized controlled trial in PREVIEW: the addressees (pre-diabetics), the intervention objective (the prevention of T2D) and the treatment conditions (randomized-multicenter-intervention trial; supervised behavior change intervention by trained staff, but adaptable to the particular conditions of each center). PREMIT is supported by a toolkit that promotes a high fidelity, which is—besides other criteria—an important output variable in implementation research (Proctor et al., 2009). PREMIT is delivered to the onsite-instructors in form of a matrix summarizing the overall objectives of each behavior modification phase in PREVIEW as well as several sub-objectives and how they could be achieved.

Applying PREMIT will also lead to research results. As described in Step 4, PREMIT will be evaluated by a longitudinal research design. The most important research question will be: Does the behavior modification intervention lead to the intended changes in the behavioral mechanisms and, subsequently, in the behavior itself. Effectiveness of the behavior change program will be analyzed by a repeated measurements design at six measurement points (using the questionnaires indicated in Step 4). Study participants' physical activity volume as well as their dietary behavior will also be measured at these six measurement points by using diaries and (for physical activity only) repeated accelerometer measurements. The questionnaire data are collected online via a Questionnaire Delivery Platform and stored in a central data hub at the University of Copenhagen, where the questionnaire data are merged with data from the accelerometer.

Above all descriptive analyses, which will be applied, the Multiple Latent Change Score Modeling Approach (MLCSM; McArdle, 2009) will mainly be used to analyze the associations between the behavioral mechanisms (i.e., latent factors), their hypothesized and assessed changes over time and the intended and registered behavioral changes. MLCSM uses data from baseline and following measurement points in order to provide information on intra-individual as well as inter-individual differences in changes of latent factors. Physical activity volume and dietary behavior will be the dependent variables (i.e., manifest variables). MLCSM models will be used to test the assumptions (a) if changes in behavioral mechanisms occurred as intended and (b) whether these changes predict changes in participants' behavior. Further, it is hypothesized that (c) behavioral mechanisms vary upon their impact on behavior throughout the process of behavior change. For instance, as postulated by the Health Action Process Approach (Schwarzer, 1992) outcome expectancies should impact participants' behavior during the first stages of behavior change (i.e., the LCD and preparation stage) whereas coping self-efficacy should impact participants' behavior during later stages (i.e., action stage, maintenance stage). Statistical analyzes will be conducted using Mplus.

## Discussion

PREMIT guides the PREVIEW intervention and will be used to investigate the dynamic processes of a sustainable behavior change process. As the intervention occurs in a multi-center study and in a natural setting, it is faced with some challenges.

One challenge is the implementation of the program in six different European countries, which were chosen to represent North, South, West and East Europe as well as in Australia and New Zealand. Centers have different traditions, cultural norms, facilities and resources and local and national policies. To this end, care was taken to assure that the effectiveness of PREMIT was independent of the center as well as the professions and experiences of the site instructors. In order to reach independence, materials, SOP and participant instructions serve as means for standardization. On the other hand, each site will also adapt PREMIT to suit their population (i.e., cultural traditions and norms) and context. Ultimately each center will fine-tune PREMIT according to their national, regional or local features (e.g., such as cultural norms and preferences). Moreover, behavior change fails if cultural norms and situation specific circumstances are ignored.

A further challenge concerns the group visits. Those arrangements are a common and cost-effective organizational structure to counsel people (compared to an individual counseling). At the end of PREVIEW, observations will identify to what extent a group setting fitted to the participants needs. For example, some people may pass faster through the PREMIT stages or react in different ways to the procedures than others or they would prefer being in an individual counseling program. Counselors were also expected to use PREMIT approaches flexibly and intuitively depending on the needs of participants during group visits.

In conclusion, a significant investment was required to develop PREMIT. Several experts from different project partners were involved. Substantial time was spent developing and adapting the toolkit to fit study requirements, participants' needs and scientific requirements and cultural diversity. For instance, the need for detailed descriptions and standardized procedures took several weeks. Further, questionnaires were translated and back translated into five different languages. In addition to these economic issues, a RE-AIM (Glasgow et al., 2001) analysis will be done in order to evaluate the conditions required to implement the intervention successfully.

PREMIT is, with its strict definition of psychological mechanisms, expected to influence physical activity and nutrition behavior, using a theoretical framework that promotes behavior change in people at risk of T2D in a natural setting. PREMIT uses behavior change theories and principles explicitly and aims to report the intervention program transparently. At the end of the 3 years PREVIEW trial, the study group will have increased their knowledge about the effectiveness of a long lasting behavior change regimen in participants at risk of T2D and informs the understanding of implementation science in a real world intervention.

## Author contributions

The PREMIT program was conducted and developed by DK, AU, and WS with contributions from GS, TM, MF. MF, AR, TM, WS, GS conducted the overall study protocol. AR, MF, WS, contributed to obtain funding for the PREVIEW project. DK, AU, WS drafted the manuscript. The manuscript was submitted while DK was employed at the University of Stuttgart. GS, TM, MF, AR reviewed the manuscript and provided comments and revision. All authors approved the final manuscript.

### Conflict of interest statement

The authors declare that the research was conducted in the absence of any commercial or financial relationships that could be construed as a potential conflict of interest.

## Footnotes

^1^In the meantime, the taxonomy has been extended (Michie et al., 2013), However, we developed the PREMIT intervention between October 2012 and March 2013, where the CALO-RE taxonomy was already available.

## Supplementary material

Supplementary Table 1 **Overview of behavior change stages, objectives, behavioral mechanisms/predictors, behavior change techniques and tools, as well as assessments**.

## Abbreviations

BCT: behavior change technique
BMI: Body Mass Index
LCD: Low calorie diet
PREMIT: PREview behavior Modification Intervention Toolbox
PREView: Prevention of diabetes through lifestyle Intervention and population study in Europe and around the World
RCT: Randomized controlled trial
SOP: Standard operational procedure
T2D: Type-2 diabetes mellitus.
